# Platelet-derived growth factor receptor-β as a non-invasive biomarker for liver fibrosis prediction in Egyptian diabetic patients with metabolic-associated fatty liver disease

**DOI:** 10.1186/s12876-024-03542-y

**Published:** 2025-07-17

**Authors:** Hanaa Badran, Maha Elsabaawy, Mai Magdy, Hazem Omar, Olfat Hendy, Eman Awaad, Maymona Abd El-Wahed Al-Khalifa, Mai Abozeid

**Affiliations:** 1 Hepatology and Gastroenterology Department, National Liver Institute, Menoufia University, Shebin El-Kom, Menoufia, 32511 Egypt; 2 Diagnostic and Interventional Radiology and Medical Imaging Department, National Liver Institute, Menoufia University, Shebin El-Kom, Menoufia, Egypt; 3 Clinical and Chemical Pathology Department, National Liver institute, Menoufia University, Shebin El-Kom, Menoufia, Egypt; 4 Anesthesiology and ICU Department, National Liver institute, Menoufia University, Shebin El-Kom, Menoufia, Egypt; 5 Fellow Lecturer in Therapeutic Nutrition Department, National Liver institute, Menoufia University, Shebin El-Kom, Menoufia, Egypt

**Keywords:** Liver fibrosis, Diabetes mellitus, MAFLD, PDGFRβ

## Abstract

**Background:**

Circulating platelet-derived growth factor receptor-β (PDGFRβ) has recently been found to correlate with severity of liver disease in multiple etiologies, including liver steatosis. In diabetic patients with metabolic-associated fatty liver disease (MAFLD), widely used non-invasive scoring systems, particularly the fibrosis-4 (FIB-4) score, showed unsatisfactory performance in predicting liver fibrosis severity. The aim of this study was to evaluate the productivity of serum PDGFRβ as a non-invasive biomarker of liver fibrosis in diabetic MAFLD patients.

**Methods:**

This was a population-based case-control study conducted on 50 diabetic MAFLD patients, 40 nondiabetic MAFLD patients, and 40 healthy controls. All subjects underwent complete history taking, clinical examination, anthropometric measurements, bioelectrical impedance analysis (BIA), and laboratory tests, including the PDGFRβ assay. Hepatic steatosis was assessed with magnetic resonance imaging (MRI), along with magnetic resonance elastography (MRE) for the assessment of liver fibrosis. The diagnostic performance of PDGFRβ as well as PDGFRβ + FIB-4 in prediction of significant liver fibrosis in diabetic MAFLD patients was assessed.

**Results:**

Liver steatosis and significant liver fibrosis (≥ F2) were significantly higher in diabetic MAFLD patients than in nondiabetics. PDGFRβ levels were significantly higher in both diabetic and nondiabetic MAFLD patients compared to controls. Sensitivity, specificity, positive predictive value (PPV), and negative predictive value (NPV) of PDGFRβ to predict significant liver fibrosis in diabetic MAFLD patients were 85%, 93.33%, 89.5%, and 90.3%, respectively, at a cutoff > 2.54, and were 85.71%, 51.52%, 27.3%, and 94.4% at a cutoff > 1.59 in nondiabetics. Sensitivity, specificity, PPV, and NPV of (PDGFRβ at a cutoff > 2.54 + FIB-4 at a cutoff > 1.17) to predict significant liver fibrosis in diabetic MAFLD patients were 100%. PDGFRβ was the only independent predictor of significant liver fibrosis in diabetic MAFLD (*p* = 0.006).

**Conclusions:**

PDGFRβ proved efficacy as a noninvasive biomarker in the prediction of significant liver fibrosis (≥ F2) in diabetic MAFLD patients.

## Background

One of the most prominent causes of chronic liver disease nowadays is nonalcoholic fatty liver disease (NAFLD), or as recently termed metabolic-associated fatty liver disease (MAFLD), affecting 25–30% of the global population. This high prevalence of MAFLD has been proposed to parallel the worldwide rising rates of obesity and type 2 diabetes mellitus (T2DM) [1]. NAFLD was defined by the presence of steatosis in > 5% of hepatocytes in the absence of significant ongoing or recent alcohol consumption and other known causes of liver disease. MAFLD more appropriately characterizes the liver disease linked to known metabolic dysfunction. The new nomenclature defines a set of positive criteria, rather than exclusion criteria, independent of the amount of alcohol consumed and without necessitating ruling out other concomitant liver diseases. MAFLD diagnosis is based on histological, imaging, or blood biomarker evidence of hepatic steatosis in addition to overweight/obesity, T2DM, or evidence of metabolic dysregulation [2].

MAFLD frequently progresses and is linked to serious consequences such as cirrhosis, hepatocellular cancer, and mortality. Liver fibrosis is the main determinant of MAFLD progression and the strongest predictor for liver-related and overall morbidity and mortality [3]. A liver biopsy is the gold standard for assessing hepatic fibrosis. However, because it is invasive and comes with risks such as cost, sampling error, and interobserver variability, it is neither practical nor desirable to be performed for every patient with suspected MAFLD. So, there is an urgent demand for trustworthy, precise, non-invasive, or minimally invasive biomarkers [4]. The least invasive methods that are frequently used are liver stiffness, composite scores, and plasma biomarkers [5].

There is a known higher risk of MAFLD, metabolic-associated steatohepatitis (MASH), and advanced fibrosis in patients with T2DM. Therefore, a variety of guidelines recommend widespread screening of these patients using non-invasive methods [6]. A lot of studies have conflicting data regarding the performance of non-invasive tests in MAFLD, especially in diabetics. A recent survey in a global MAFLD consensus statement from 24 countries revealed that cut-offs used for the same test vary between clinicians [7].

Notably, the majority of research on noninvasive testing in MAFLD has not taken diabetes into account while conducting its analyses. According to a number of studies, noninvasive tests that were approved in cohorts without diabetes performed poorly in diabetic patients [8]. It was confirmed that commonly used fibrosis scores had reasonable specificity but poor PPV for detecting significant and advanced fibrosis in diabetics with MAFLD [9–12]. These patients may have a different metabolic and biochemical fingerprint compared with MAFLD patients without T2DM. So, development of reliable biomarkers for MAFLD/MASH in diabetics is urgently needed.

PDGFRβ can be a promising biomarker to detect significant liver fibrosis in diabetic MAFLD patients. In stellate cells, PDGFRβ signaling is the most prominent mitogenic pathway. In a healthy liver, PDGFRβ expression is minimal, but when the liver is injured, it substantially increases in stellate cells. It is thus a biomarker of fibrogenesis in the liver [13]. Onogi et al. found that the systemic deletion of PDGFRβ inhibited neovessel formation in adipose tissues, reduced fat accumulation and inflammation in visceral adipose tissue, and improved glucose metabolism with decreased ectopic fat deposition in the muscle and liver of obese mice [14]. PDGFRβ-targeted positron emission tomography (PET) has a great potential as an imaging biomarker for assessing disease progression and treatment responses in multiple liver fibrosis etiologies, including viral hepatitis (HBV, HCV) [15]. According to recent research, stromal cell behavior, particularly that induced by PDGFRβ, is associated with inflammation, regeneration, and malignancy in addition to the pathogenesis of fibrosis. The PDGFRβ pathway has a major role in the development and progression of fibrosis in T2DM and its complications [16]. On the other hand, little is known about the application of circulating levels of PDGFRβ as a diagnostic tool, especially in diabetic MAFLD patients [17]. Therefore, we examined the productivity of serum PDGFRβ as a non-invasive biomarker of liver fibrosis in diabetic MAFLD patients.

## Methods

This population-based case-control study was conducted at the Hepatology and Gastroenterology Department, National Liver Institute, Menoufia University, Egypt, on 50 MAFLD patients with T2DM. In addition, 40 non-diabetic MAFLD patients, as well as 40 healthy individuals as controls, with matched sex and age, were enrolled. The sample size was calculated using the OpenEpi website (http://www.openepi.com/SampleSize/SSCC.htm). The study was approved by the National Liver Institute Ethics Committee (Protocol number 00227/2020). After each participant provided informed consent, the following parameters were assessed:

- Sociodemographic data: including age, gender, occupation, education, residence, and life style.
- Clinical data: including physical examination, vital signs (blood pressure, temperature, heart rate, and respiratory rate), anthropometric measurements (weight, height, body mass index (BMI) (kg/m2), waist circumference (cm), hip circumference (cm), and waist/hip circumference ratio), alcohol and tobacco use, contraception, as well as the existence of metabolic risk factors (obesity, dyslipidemia, hypertension).
- Therapeutic data: including concomitant and/or previous medical treatment.
- Paraclinical data:
  - Laboratory characteristics: including liver function tests (Aspartate transaminase (AST), Alanine transaminase (ALT), International normalized ratio (INR), albumin), complete blood count (hemoglobin, total leucocyte count (TLC), platelets), creatinine level, lipid profile (total cholesterol, high density lipoprotein (HDL), low density lipoprotein (LDL), triglycerides), blood glucose, HbA1c, fasting insulin, anti-HCV, HbsAg, and anti-HBc). HOMA-IR was calculated as [(fasting glucose x fasting insulin)/405] [18].
  - Imaging characteristics: including pelviabdominal ultrasound, MRI, and MRE findings.

### The control group

Included healthy adult volunteers with normal clinical, laboratory, and imaging parameters. MAFLD was excluded by the absence of: hepatic steatosis on imaging; related comorbidities; or any metabolic risk abnormalities.

### Inclusion criteria for patient groups

Adult (> 18 years old) males and females with or without T2DM who met MAFLD diagnostic criteria [19]. MAFLD was diagnosed by the presence of a bright, fatty liver on ultrasound and liver steatosis by MRI plus overweight or obesity, T2DM, or ≥ 2 metabolic risk abnormalities including a waist circumference of ≥ 102/88 cm in Caucasians (or ≥ 90/80 cm in Asians), a blood pressure of ≥ 130/85 mmHg or receiving a specific drug treatment, plasma triglycerides of ≥ 150 mg/dL (≥ 1.70 mmol/L) or receiving a specific drug treatment, plasma HDL cholesterol of < 40 mg/dL (< 1.0 mmol/L) in men and < 50 mg/dL (< 1.3 mmol/L) in women or receiving a specific drug treatment, prediabetes (i.e., fasting glucose levels of 100–125 mg/dL (5.6–6.9 mmol/L), or 2-hours post-load glucose levels of 140–199 mg/dL (7.8–11.0 mmol), or HbA1c of 5.7–6.4%), HOMA-IR of ≥ 2.5, and plasma hs-CRP levelof > 2 mg/L.

Diabetes mellitus type 2 was diagnosed as a history of T2DM or one or more of the modified International Diabetes Federation and World Health Organization diagnostic criteria: fasting blood sugar ≥ 126 mg/dl or two-hour plasma glucose after 75 g oral glucose load ≥ 200 mg/dl or HbA1c ≥ 6.5% or random blood sugar ≥ 200 mg/dl [20].

### Exclusion criteria for patient groups

Age < 18, ≥ 10% weight loss within 6 months, subjects with other causes of liver disease, consuming alcohol, or receiving steatogenic drugs. Patients with hepatic decompensation, hepatic encephalopathy, ascites, variceal bleeding, kidney diseases, autoimmune diseases, thyroid abnormalities, malignancy, or sepsis were also excluded.

### Bioelectrical Impedance Analysis (BIA)

The multi-frequency bioelectrical impedance analyzer (Tanita MC-780MA; Tanita, Tokyo, Japan) was used. Watches, jewelry, and belts were removed, and patients were asked to stand barefoot on the machine platform with their arms abducted and their hands gripping on to the machine handle. All the data obtained were normalized for age, sex, and height. Body fat mass is expressed in kg and as a percentage of body weight. Visceral fat ranges from 1 to 59 (1–12: healthy level, 13–59: excessively unhealthy level) [21].

## Assessment of non-invasive hepatic fibrosis scores

The AST/ALT ratio [22], FIB-4 index [23], APRI [24], King’s score [25], NAFLD fibrosis score [26], and Hepamet fibrosis score [27] were calculated for diabetic and nondiabetic MAFLD patients.

## Hepatic steatosis evaluation with magnetic resonance imaging (MRI)

All patients were subjected to MR (in-phase and out-phase) using a 1.5-T MR imaging system (1.5-T Optima 450 W GEM Suite, GE, USA). Patients were fasting for six hours before the examination. An integrated body coil and a linear general-purpose flexible surface coil were used. A coronal scout image of the abdomen was obtained. In-phase and out-of-phase transverse T1-weighted dual-echo fast spoiled-gradient-recalled images of the entire liver were obtained during breath holding. Imaging parameters were as follows: repetition time 75–100 msec; echo time msec: 2.3 (out of phase) or 4.6 (in phase); 7–8 mm section thickness; and 23-second acquisition time.

Unaware of the clinical information, a radiologist evaluated the extent of hepatic steatosis on a photoarchive and communication system workstation using qualitative and quantitative methods. The region of interest was drawn so as to be a 2 cm^2^ area without blood vessels or an artifact and was matched to the same location for each sequence. An equation was used to calculate fat indices (FI) from signal intensity (SI) differences between in-phase and out-of-phase images (**FI = (SI**_**in**_**– SI**_**out**_**) ÷ (SI**_**in**_**) x 100**). Steatosis grades were reported according to the FI equation: grade 0 (minimal steatosis) ≤ 5%, grade 1 (mild) > 5–≤ 33%, grade 2 (moderate) > 33–≤ 66%, and grade 3 (severe) > 66% [28].

## Liver stiffness evaluation with magnetic resonance elastography (MRE)

Patients underwent the standard hepatic MRI protocol with the 1.5-T MR imaging system (1.5-T Optima 450 W GEM Suite, GE, USA). Mechanical shear waves were created through longitudinal, continuous vibrations of 60 Hz transmitted from the active driver to the passive driver. These waves were recorded using a specific MRI pulse sequence that had synchronized motion sensitizing gradients intended to image the generating shear wave image (measured in micrometers (µm)) and the micron-level cyclic displacements brought on by the propagating waves.

Automated processing of obtained data, with an inversion algorithm, created elastogram images for estimation of tissue stiffness in Kilopascal (kPa). Liver stiffness measurement (LSM) was carried out by drawing regions of interest (ROIs) on the elastograms. ROIs include areas of the liver with enough wave amplitude and away from edge effects (at least half a wave length away from the liver border), the gallbladder fossa, large vessels, as well as any areas affected by cardiac and vascular artifacts. The mean ROIs were averaged and reported as the mean LSM of the liver. The cutoffs for stage 1, stage 2, stage 3, and stage 4 fibrosis were 2.88, 3.54, 3.77, and 4.09 kPa, respectively [29].

## Platelet derived growth factor receptor-β (PDGFRβ) assay

Serum PDGFRβ levels were assessed using enzyme-linked immunosorbent assay (ELISA) kits (PDGFRB Primacu™, BT LAB, Zhejiang, China), as instructed by the manufacturer [30]. Serum samples as well as all reagents and standards were prepared according to the instructions. The sample and ELISA reagent were added into each well and incubated for 1 h at 37 °C. After that, wash buffer was used to wash the plate five times. Substrate solutions A and B were then added to each well, and the wells were incubated at 37 °C for 10 min. Stop Solution was then added to each well, and the color changed immediately. After adding the stop solution, each sample was immediately measured for optical density (OD) using a microplate reader set to 450 nm in less than ten minutes. Using computer-based curve-fitting software, a standard curve was created for the results computation by graphing the average OD for each standard on the vertical (Y) axis against the concentration on the horizontal (X) axis. Regression analysis was then used to identify the best fit line.

### Statistical analysis

We analyzed the data using the statistical package of the social sciences (SPSS 22.0, IBM/SPSS Inc., Armonk, USA). The Shapiro-Wilk test was used to define the normality of the distribution of different variables. The chi-square test was employed for categorical variables to compare groups. For quantitative data, to compare between more than two groups, the Kruskal-Wallis test was used for abnormally distributed continuous variables, and the F-test (ANOVA) was used for normally distributed continuous variables, followed by the post-hoc test for pair-wise comparisons. To compare between two independent groups, the Mann-Whitney test was applied for abnormally distributed continuous variables, and the Student t test was used for normally distributed continuous variables. The Spearman coefficient was utilized for correlation analysis. The receiver operating characteristic curve (ROC) was employed to denote the diagnostic performance of the test (sensitivity, specificity, positive and negative predictive value, as well as accuracy) and for a comparison of performance between tests. The cutoff values for PDGFRβ were selected according to the best performance (sensitivity and/or specificity). The univariate and multivariate logistic regression methods were performed to predict a dependent data variable. A p value of less than 0.05 was established for statistical significance.

## Results

Diabetic MAFLD patients had the highest frequency of hypertension and metabolic syndrome compared to nondiabetics as well as controls (*p* < 0.001) (Table 1). BMI was significantly higher in the diabetic and nondiabetic MAFLD patients in comparison to the control group (37.22 ± 6.84 and 35.13 ± 5.60 vs. 23.38 ± 1.77, respectively) (*P* < 0.001). In females, waist circumference, hip circumference, and waist/hip ratio were significantly higher in both MAFLD groups compared to controls (117.82 ± 11.54, 125.9 ± 11.7, and 0.94 ± 0.05 in diabetics and 115.6 ± 12.12, 124.61 ± 11.25, and 0.93 ± 0.07 in nondiabetics vs. 86.85 ± 4.42, 105.65 ± 4.4, and 0.82 ± 0.02 in controls, respectively) (*p* < 0.001). In males, they were significantly higher in the diabetic MAFLD group compared to controls (112.09 ± 15.57, 116.64 ± 11.74, and 0.95 ± 0.1 vs. 92.14 ± 6.35, 106.36 ± 4.2, and 0.87 ± 0.05, respectively) (*p* = 0.001, 0.022, and 0.024, respectively). The bioelectrical impedance analysis (BIA) showed that visceral fat was significantly higher in diabetic MAFLD patients than in nondiabetics (13.12 ± 4.01 vs. 11.18 ± 3.24, *p* = 0.026). Also, it was significantly higher in the diabetic and nondiabetic MAFLD patients compared to controls (13.12 ± 4.01 and 11.18 ± 3.24 vs. 8.45 ± 2.97, respectively) (*P* < 0.001). Significantly higher values of fat percentages were detected in the diabetic and nondiabetic MAFLD groups in comparison with controls (40.05 ± 8.83 and 37.71 ± 8.16 vs. 24.47 ± 8.11, respectively) (*P* < 0.001). Similarly, the fat mass was significantly higher in the diabetic and nondiabetic MAFLD patients compared to controls (38.19 ± 12.28 and 36.09 ± 10.26 vs. 18.34 ± 7.41, respectively) (*P* < 0.001) (Table 2).

**Table 1:** Clinical criteria of the studied groups

	Diabetic MAFLD (*N* = 50)		Nondiabetic MAFLD (*N* = 40)		Controls (*N* = 40)		Test	*p*	Sig. between Groups		
									*p*_1_	*p*_2_	*p*_3_
Age (years)											
• Min. – Max.	39–60		39–60		38–60		**F**	0.161			
• Mean ± SD.	51.46 ± 7.54		48.40 ± 7.47		49.55 ± 7.95						
	**N**	**%**	**N**	**%**	**N**	**%**					
Sex											
• Male	11	22	9	22.5	14	35	**χ2**	0.310			
• Female	39	78	31	77.5	26	65					
Smoking											
• No	44	88	37	92.5	35	87.5	**χ2**	0.824			
• Yes	6	12	3	7.5	5	12.5					
Hypertension											
• No	28	56	29	72.5	40	100	**χ2**	**< 0.001** ^*^	0.107	**< 0.001** ^*^	**< 0.001** ^*^
• Yes	22	44	11	27.5	0	0					
Metabolic syndrome											
• No	6	12	21	52.5	40	100	**χ2**	**< 0.001** ^*^	**< 0.001** ^*^	**< 0.001** ^*^	**< 0.001** ^*^
• Yes	44	88	19	47.5	0	0					

N: number, Mean ± SD: (mean ± standard deviation), Min. – Max: minimum-maximum, F: ANOVA test, χ2: Chi square test, p: p value, p_1_: diabetic vs. nondiabtic, p_2_: diabetic vs. controls, p_3_: nondiabetic vs. controls, *: Statistically significant at *p* < 0.05

**Table 2:** Anthropometric measurements and bioelectrical impedance analysis (BIA) of the studied groups

			Diabetic MAFLD (*N* = 50)	Nondiabetic MAFLD (*N* = 40)	Controls (*N* = 40)	Test	*p*	Sig. bet. Groups		
								*p*_1_	*p*_2_	*p*_3_
Anthropometric measurements										
• Waist circumference (cm):										
Male	Min. – Max.	77 – 132		77 – 119	79–99	**F**	**0.001** ^*^	0.317	**0.001** ^*^	0.067
	Mean ± SD.	112.09 ± 15.57		104.1 ± 13.91	92.14 ± 6.35					
Female	Min. – Max.	92 – 140		90 – 140	77 – 94	**F**	**< 0.001** ^*^	0.666	**< 0.001** ^*^	**< 0.001** ^*^
	Mean ± SD.	117.82 ± 11.54		115.6 ± 12.12	86.85 ± 4.42					
• Hip circumference (cm):										
Male	Min. – Max.		88 – 133	97 – 131	97 – 112	**F**	**0.029** ^*^	0.359	**0.022** ^*^	0.460
	Mean ± SD.		116.64 ± 11.74	111 ± 10.71	106.36 ± 4.2					
Female	Min. – Max.		103 – 148	106 – 160	97 – 111	**F**	**< 0.001** ^*^	0.853	**< 0.001** ^*^	**< 0.001** ^*^
	Mean ± SD.		125.9 ± 11.7	124.61 ± 11.25	105.65 ± 4.4					
• Waist/hip ratio:										
Male	Min. – Max.		0.78–1.15	0.79–1.05	0.79–0.94	**F**	**0.020** ^*^	0.888	**0.024** ^*^	0.099
	Mean ± SD.		0.95 ± 0.1	0.94 ± 0.07	0.87 ± 0.05					
Female	Min. – Max.		0.84–1.04	0.77–1.06	0.79–0.91	**F**	**< 0.001** ^*^	0.884	**< 0.001** ^*^	**< 0.001** ^*^
	Mean ± SD.		0.94 ± 0.05	0.93 ± 0.07	0.82 ± 0.02					
• BMI ( Kg/m^2^):										
Min. – Max.			18.3 – 50.1	20.2–44.9	19 – 24.9	**F**	**< 0.001** ^*^	0.159	**< 0.001** ^*^	**< 0.001** ^*^
Mean ± SD.			37.22 ± 6.84	35.13 ± 5.60	23.38 ± 1.77					
Bioelectrical impedance analysis (BIA)										
V Fat	Min. – Max.		1– 20	1–18	3–12	**F**	**< 0.001** ^*^	**0.026** ^*^	**< 0.001** ^*^	**< 0.001** ^*^
	Mean ± SD.		13.12 ± 4.01	11.18 ± 3.24	8.45 ± 2.97					
Fat P (%)	Min. – Max.		4.2–52	18.2–48.4	4.2–34	**F**	**< 0.001** ^*^	0.392	**< 0.001** ^*^	**< 0.001** ^*^
	Mean ± SD.		40.05 ± 8.83	37.71 ± 8.16	24.47 ± 8.11					
Fat M (Kg)	Min. – Max.		2.4–60.2	11.9–55.7	2.4–36	** F**	**< 0.001** ^*^	0.606	**< 0.001** ^*^	**< 0.001** ^*^
	Mean ± SD.		38.19 ± 12.28	36.09 ± 10.26	18.34 ± 7.41					

N: number, M ± SD: mean ± standard deviation, Min. – Max: minimum-maximum, BMI: body mass index, V fat: visceral fat, Fat P: fat percentage, Fat M: Fat mass, F: one way ANOVA test (Post Hoc Test (Tukey) for pairwise comparison), p: p value, p_1_: diabetic vs. nondiabtic, p_2_: diabetic vs. controls, p_3_: nondiabetic vs. controls, *: Statistically significant at *p* < 0.05

## PDGFRβ and laboratory characteristics of the studied groups

PDGFRβ levels were significantly higher in both MAFLD subgroups compared to controls. Significantly higher AST and ALT values were found in diabetic and nondiabetic MAFLD patients than in the control group. Diabetic MAFLD patients exhibited a significantly reduced serum albumin level compared to controls. Total cholesterol, low-density lipoprotein (LDL), and HOMA IR were significantly higher in diabetic MAFLD patients in comparison to nondiabetic patients as well as controls. Triglycerides were significantly higher in MAFLD subgroups than in controls and at the verge of significance (*p* = 0.054) between diabetic and nondiabetic MAFLD patients. HDL significantly differed between the diabetic MAFLD group and controls (*p* = 0.022). Insulin levels were significantly higher in both MAFLD subgroups than in the control group, but there was no significant difference between diabetic and nondiabetic MAFLD patients (Table 3).

**Table 3:** PDGFRβ levels and laboratory characteristics of the studied groups

		Diabetic MAFLD (*N* = 50)	Nondiabetic MAFLD (*N* = 40)	Controls (*N* = 40)	Test	p	Sig. bet. Groups		
							**p**_**1**_	**p**_**2**_	**p**_**3**_
PDGFR β (ng/mL)	Min. – Max.	1.14–8.30	1.07–5.60	0.97–4.30	**H**	**0.007** ^*^	0.525	**0.002** ^*^	**0.022** ^*^
	Mean ± SD.	2.57 ± 1.77	2.24 ± 1.23	1.64 ± 0.64					
AST (U/L)	Min. – Max.	11–79	14–95	12–28	**H**	**< 0.001** ^*^	0.296	**< 0.001** ^*^	**< 0.001** ^*^
	Mean ± SD.	28.4 ± 12.68	31.13 ± 21.5	18.95 ± 3.97					
ALT (U/L)	Min. – Max.	11–112	12–130	11–31	**H**	**< 0.001** ^*^	0.172	**0.005** ^*^	**< 0.001** ^*^
	Mean ± SD.	29.88 ± 19.03	37.70 ± 26.13	20.23 ± 6.42					
Albumin (g/dl)	Min. – Max.	3.6–4.8	3.7–5.3	3.9–5	**F**	**0.015** ^*^	**0.250**	**0.011** ^*^	0.415
	Mean ± SD.	4.25 ± 0.33	4.36 ± 0.29	4.45 ± 0.33					
INR	Min. – Max.	0.89–1.20	0.89–1.10	0.89–1.1	**F**	0.112			
	Mean ± SD.	1.02 ± 0.09	1 ± 0.05	0.99 ± 0.09					
Hemoglobin (g/dl)	Min. – Max.	9.2–15.9	11.3–16.1	11.20–17.2	**F**	0.051			
	Mean ± SD.	12.96 ± 1.45	13.39 ± 1.02	13.56 ± 1.02					
TLC (*10^3^/UL)	Min. – Max.	4.1–15.5	4.20–13.4	2.11–10.2	**H**	0.239			
	Mean ± SD.	7.24 ± 2.21	6.52 ± 1.72	6.74 ± 1.99					
Platelets (*10 ^3^/UL)	Min. – Max.	157– 425	191– 578	153– 484	**H**	0.293			
	Mean ± SD.	250.92 ± 59.34	269.38 ± 68.03	256.63 ± 99.07					
Total cholesterol (mg/dl)	Min. – Max.	153–351	148–382	142–220	**H**	**< 0.001** ^*^	**0.003** ^*^	**< 0.001** ^*^	**0.007** ^*^
	Mean ± SD.	253.54 ± 55.92	221.05 ± 59.86	187.48 ± 19.08					
HDL (mg/dl)	Min. – Max.	29– 73	28–72	29–105	**F**	**0.019** ^*^	0.956	**0.022** ^*^	0.063
	Mean ± SD.	46.21 ± 9.19	46.88 ± 11.12	52.45 ± 12.70					
LDL (mg/dl)	Min. – Max.	66.6–275	80.2 − 280.4	72.4–129.1	**H**	**< 0.001** ^*^	**0.003** ^*^	**< 0.001** ^*^	**0.001** ^*^
	Mean ± SD.	177.04 ± 52.39	145.16 ± 50.72	109.92 ± 16.71					
Triglycerides (mg/dl)	Min. – Max.	69–247	69–459	48–150	**H**	**< 0.001** ^*^	0.054	**< 0.001** ^*^	**0.016** ^*^
	Mean ± SD.	151.46 ± 44.39	145.08 ± 64.61	112.25 ± 30.77					
Insulin (mIU/L)	Min. – Max.	4.09–28.49	3.75–23.94	3.18–13.54	**H**	**< 0.001** ^*^	0.150	**< 0.001** ^*^	**0.001** ^*^
	Mean ± SD.	14.48 ± 6.87	12.37 ± 5.35	8.57 ± 2.48					
HOMA IR	Min. – Max.	1.30–13.61	0.88–6.09	0.64–2.4	**H**	**< 0.001** ^*^	**0.005** ^*^	**< 0.001** ^*^	**0.001** ^*^
	Mean ± SD.	5.38 ± 3.34	2.89 ± 1.26	1.87 ± 0.57					

N: number, M ± SD mean ± standard deviation, Min. – Max: minimum-maximum, PDGFRβ: platelet derived growth factor receptor β. AST: Aspartate transaminase, ALT: Alanine transaminase, INR: International normalized ratio, TLC: Total leucocytes count, HDL: high density lipoprotein, LDL: low density lipoprotein, HOMA IR: homeostasis model assessment-estimated insulin resistance. H: H for Kruskal Wallis test (Post Hoc Test (Dunn’s) for pairwise comparison), F: one way ANOVA test (Post Hoc Test (Tukey) for pairwise comparison), p: p value, p_1_: diabetic vs. nondiabtic, p_2_: diabetic vs. controls, p_3_: nondiabetic vs. controls, *: Statistically significant at *p* < 0.05

### Liver steatosis by MRI, LSM by MRE, and fibrosis stages in MAFLD subgroups

Diabetic MAFLD patients had a significantly higher degree of liver steatosis by MRI compared to nondiabetics (*p* = 0.007). LSM by MRE was significantly higher (*p* = 0.038) in diabetics (mean 2.88 ± 0.81 kPa) than in nondiabetics (mean 2.46 ± 0.71 kPa). Diabetic MAFLD patients had a significantly higher percentage of significant liver fibrosis (≥ F2) than the nondiabetic MAFLD group (*p* = 0.021) (Table 4).

**Table 4:** Liver steatosis by MRI, liver stiffness measurement (LSM) by MRE and fibrosis stages in MAFLD subgroups

		Diabetic MAFLD (*N* = 50)			Nondiabetic MAFLD (*N* = 40)		Test	*p*
Liver steatosis by MRI (%)	**Min. – Max.**		6–89		5.3– 78		**U**	**0.007** ^*^
	**Mean ± SD.**		44.78 ± 23.41		32.15 ± 20.9			
LSM by MRE (kPa)	**Min. – Max.**		1.6–4.72		1.4–4		**U**	**0.038** ^*^
	**Mean ± SD.**		2.88 ± 0.81		2.46 ± 0.71			
Liver fibrosis stages			**N**	**%**	**N**	**%**		**p**
F0			23	46	25	62.5	**χ2**	0.119
F1			7	14	8	20	**χ2**	0.448
F2			11	22	6	15	**χ2**	0.399
F3			6	12	1	2.5	**χ2**	0.094
F4			3	6	0	0	**χ2**	0.251
> F2			30	60	33	82.5	**χ2**	**0.021** ^*^
≥ F2			20	40	7	17.5		

N: number, M ± SD mean ± standard deviation, Min. – Max: minimum-maximum, LSM: liver stiffness measurement, MRI: magnetic resonance imaging, MRE: magnetic resonance elastography, kPa: kilopascal, U: Mann Whitney test, χ2: Chi square test, p: p value, *: Statistically significant at *p* < 0.05

### Non-invasive scores of liver fibrosis in MAFLD subgroups

AST/ALT ratio, NAFLD fibrosis score, FIB-4 index and Hepamet fibrosis score, and PDGFRβ + FIB-4 were significantly higher in diabetic MAFLD patients compared to the nondiabetics, while both APRI and King’s score had no significant difference between MAFLD subgroups (Table 5).

**Table 5:** Non-invasive scores of liver fibrosis in MAFLD subgroups

	Diabetic MAFLD (*N* = 50)	Nondiabetic MAFLD (*N* = 40)	Test	*p*
AST /ALT ratio				
• Min. – Max.	0.48–2.45	0.51–1.5	**t**	**0.009** ^*^
• Mean ± SD.	1.08 ± 0.42	0.87 ± 0.21		
APRI				
• Min. – Max.	0.07–1.1	0.09–1.06	**U**	0.252
• Mean ± SD.	0.31 ± 0.2	0.29 ± 0.19		
Kings score				
• Min. – Max.	1.6–18.99	2.16–20.97	**U**	0.511
• Mean ± SD.	5.94 ± 3.09	6.10 ± 4.11		
NAFLD fibrosis score				
• Min. – Max.	-10.27–4.48	-2.47–2.37	**U**	**< 0.001** ^*^
• Mean ± SD.	1.60 ± 2.94	0.53 ± 0.96		
FIB-4				
• Min. – Max.	0.37–2.05	0.40–1.63	**t**	**< 0.001** ^*^
• Mean ± SD.	1.21 ± 0.44	0.90 ± 0.27		
Hepamet fibrosis score				
• Min. – Max.	0.02–0.40	0.0–0.15	**U**	**< 0.001** ^*^
• Mean ± SD.	0.15 ± 0.11	0.03 ± 0.03		
PDGFRβ + FIB-4				
• Min. – Max.	1.51–9.90	1.75–7.23	**U**	**0.023** ^*^
• Mean ± SD.	3.99 ± 2.05	3.16 ± 1.39		

AST/ALT ratio: Aspartate transaminase/Alanine transaminase ratio, APRI: aspartate aminotransferase to platelet ratio index, FIB-4: fibrosis-4, t: Student t-test, U: Mann Whitney test, p: p value

### Correlation between LSM by MRE and non-invasive fibrosis scores in MAFLD patients

Liver stiffness by MRE was positively correlated with APRI, King’s score, NAFLD fibrosis score, FIB-4, and Hepamet fibrosis score in total MAFLD patients (*p* = 0.037, 0.035, 0.031, < 0.001, and 0.009, respectively). A positive correlation was found between LSM and FIB-4 score in both MAFLD subgroups (*p* = 0.002 and 0.032, respectively), while APRI was positively correlated with LSM in non-diabetic MAFLD only (*p* = 0.030) (Table 6).

**Table 6:** Correlation between liver stiffness measurement (LSM) by MRE and non-invasive scores of liver fibrosis

Non-invasive scores	MRE (LSM)					
	Total MAFLD patients **(** ***N*** ** = 90)**		Diabetic MAFLD **(** ***N*** ** = 50)**		Nondiabetic MAFLD **(** ***N*** ** = 40)**	
	*r*_s_	**p**	*r*_s_	**p**	*r*_s_	**p**
AST/ALT Ratio	0.031	0.770	-0.075	0.607	0.023	0.888
APRI	0.220	**0.037** ^*^	0.044	0.763	0.344	**0.030** ^*^
Kings score	0.223	**0.035** ^*^	0.180	0.210	0.216	0.180
NAFLD fibrosis score	0.227	**0.031** ^*^	0.064	0.658	0.092	0.570
FIB-4	0.451	**< 0.001** ^*^	0.433	**0.002** ^*^	0.339	**0.032** ^*^
Hepamet fibrosis score	0.273	**0.009** ^*^	0.057	0.695	0.205	0.205

LSM: liver stiffness measurement, MRE: magnetic resonance elastography, AST/ALT ratio: Aspartate transaminase/Alanine transaminase ratio, APRI: aspartate aminotransferase to platelet ratio index, FIB-4: fibrosis-4 rs: Spearman coefficient, p: p value

### Diagnostic performance of the FIB-4 score in prediction of significant liver fibrosis (≥ F2) in MAFLD patients

**Fig. 1:**
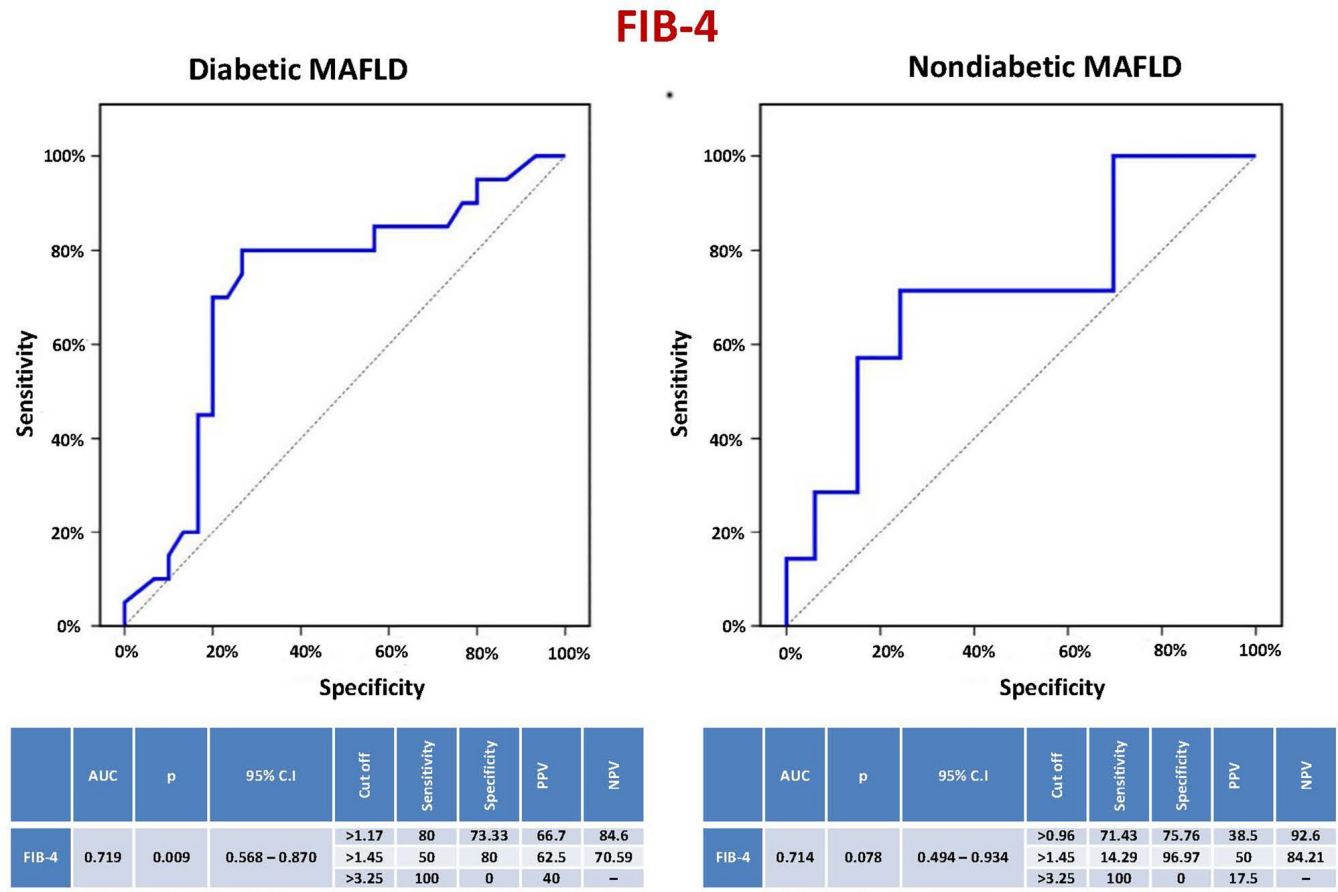
Sensitivity and specificity of the FIB-4 score to predict significant liver fibrosis in MAFLD patients. FIB-4: Fibrosis-4, MAFLD: Metabolic-associated fatty liver disease, AUC: Area under the curve, p: P value, CI: Confidence interval, PPV: Positive predictive value, NPV: Negative predictive value

The sensitivity, specificity, PPV, and NPV of the FIB-4 score to predict significant liver fibrosis (≥ F2) in diabetic MAFLD patients were 80%, 73.33%, 66.7%, and 84.6%, respectively, at a cutoff level > 1.17, while the values were 50%, 80%, 62.5%, and 70.59% at a cutoff > 1.45, and the sensitivity, specificity, and PPV at a cutoff > 3.25 were 100%, 0%, and 40%, respectively (AUC = 0.719, CI = 0.568–0.870, *P* = 0.009). In nondiabetics, they were 71.43%, 75.76%, 38.5%, and 92.6%, respectively, at a cutoff level > 0.96 and 14.29%, 96.97%, 50%, and 84.21%, respectively, at a cutoff > 1.45. At a cutoff level > 3.25, the sensitivity, specificity, and PPV were 100%, 0%, and 17.5%, respectively (AUC = 0.714, CI = 0.494–0.934, *P* = 0.078) (Fig. 1).

### Diagnostic performance of PDGFRβ in prediction of significant liver fibrosis (≥ F2) in MAFLD patients

**Fig. 2:**
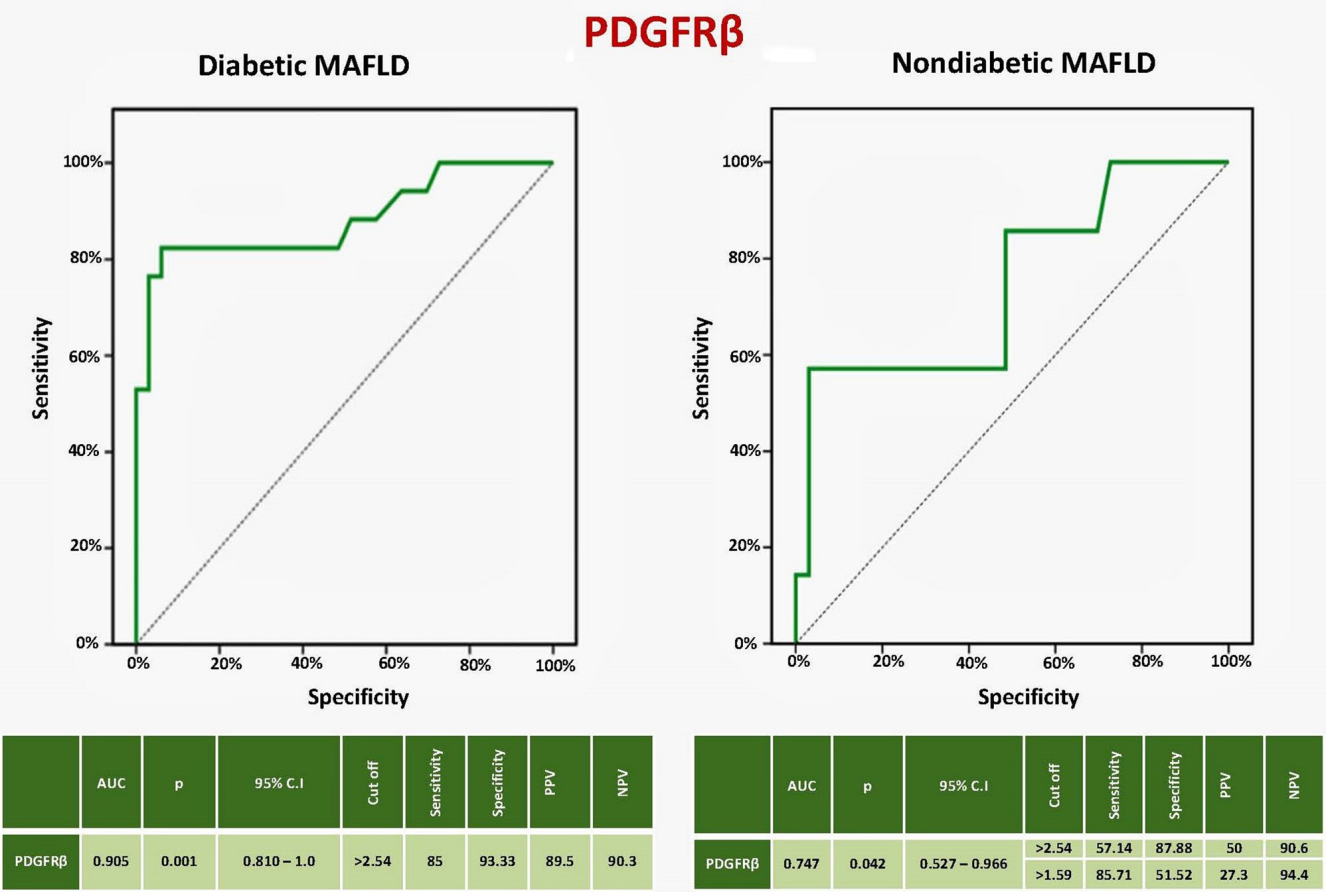
Sensitivity and specificity of PDGFRβ to predict significant liver fibrosis in MAFLD patients. PDGFRβ: Platelet-derived growth factor receptor-β, MAFLD: Metabolic-associated fatty liver disease, AUC: Area under the curve, p: P value, CI: Confidence interval, PPV: Positive predictive value, NPV: Negative predictive value

The sensitivity, specificity, PPV, and NPV of PDGFRβ to predict significant liver fibrosis (≥ F2) in diabetic MAFLD patients were 85%, 93.33%, 89.5%, and 90.3%, respectively, at a cutoff level > 2.54 (AUC = 0.905, CI = 0.810–1.0, *P* < 0.001). In the nondiabetic patients, they were 57.14%, 87.88%, 50%, and 90.6%, respectively, at a cutoff level > 2.54, while the values were 85.71%, 51.52%, 27.3%, and 94.4%, respectively, at a cutoff > 1.59 (AUC = 0.747, CI = 0.527–0.966, *P* = 0.042) (Fig. 2).

### Diagnostic performance of the PDGFRβ + FIB-4 score in prediction of significant liver fibrosis (≥ F2) in MAFLD patients

**Fig. 3:**
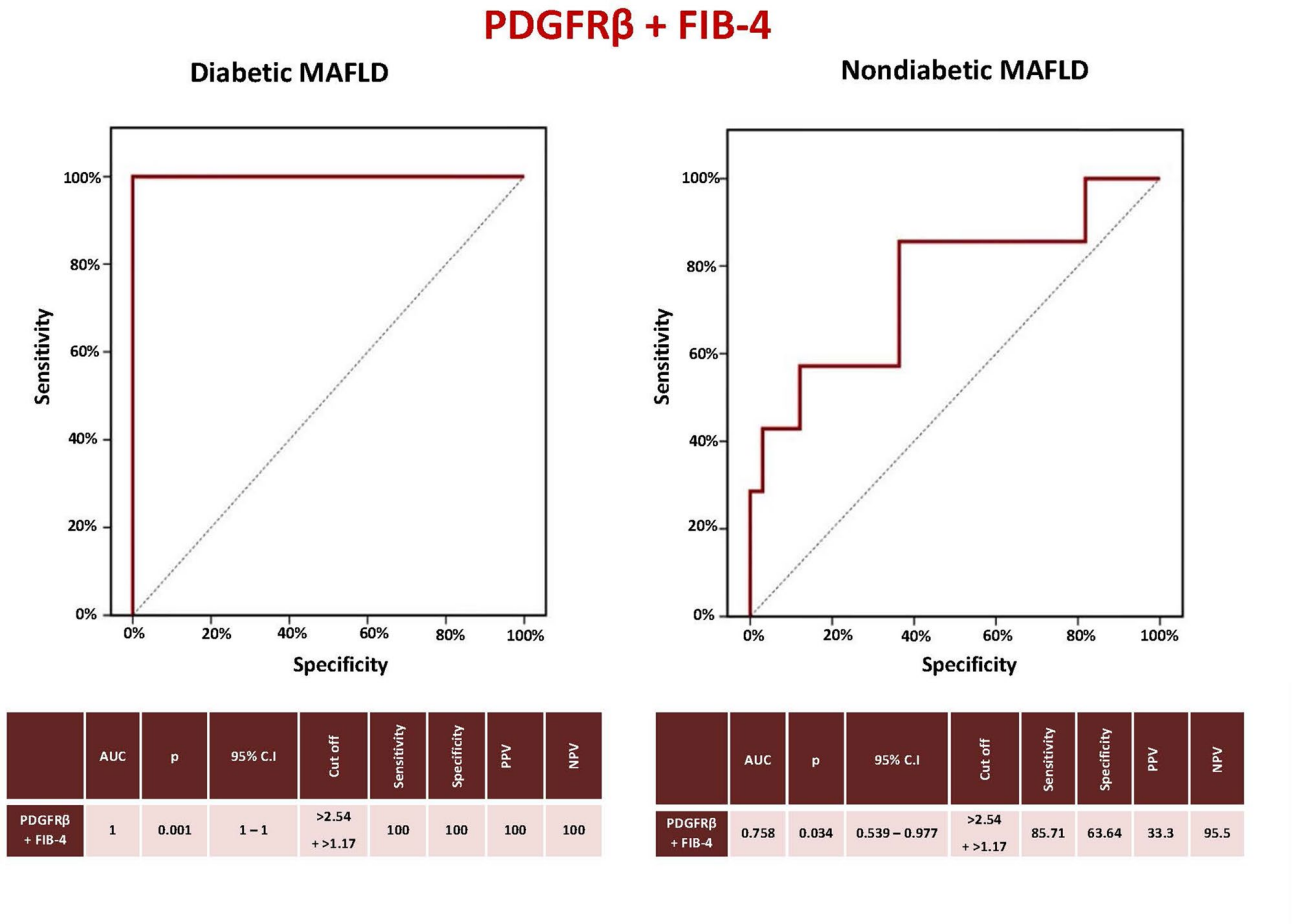
Sensitivity and specificity of PDGFRβ + FIB-4 score to predict significant liver fibrosis in MAFLD patients. PDGFRβ: Platelet-derived growth factor receptor-β, FIB-4: Fibrosis-4, MAFLD: Metabolic-associated fatty liver disease, AUC: Area under the curve, p: P value, CI: Confidence interval, PPV: Positive predictive value, NPV: Negative predictive value

The sensitivity, specificity, PPV, and NPV of PDGFRβ at a cutoff > 2.54 and FIB-4 at a cutoff > 1.07 to predict significant liver fibrosis (≥ F2) were all 100% in diabetic MAFLD (AUC = 1, CI = 1–1, *P* < 0.001). In nondiabetic NAFLD, they were 85.71%, 63.64%, 33.3%, and 95.5%, respectively (AUC = 0.758, CI = 0.539–0.977, *P* = 0.034) (Fig. 3).

### Predictors of significant fibrosis (≥ F2) in diabetic and nondiabetic MAFLD patients

Univariate logistic regression analysis revealed that factors predicting significant liver fibrosis (≥ F2) in the diabetic MAFLD group were PDGFRβ, smoking, visceral fat, and FIB-4 score (p = < 0.001, 0.047, 0.015, and 0.012, respectively). Predictors of significant fibrosis in nondiabetic MAFLD patients were PDGFRβ, smoking, and visceral fat (p = < 0.001, 0.010, and 0.048, respectively). In multivariate analysis, PDGFRβ was the only independent predictor of significant liver fibrosis (≥ F2) in diabetic MAFLD patients (*p* = 0.006) [OR (LL – UL 95% C.I) = 12.574 (2.065–76.558)] as well as in nondiabetic MAFLD patients (*p* = 0.001) [OR (LL – UL 95% C.I) = 3.313 (1.911–5.743)].

## Discussion

We enrolled 90 MAFLD patients (50 diabetic and 40 non-diabetic) as well as 40 healthy controls to assess the utility of PDGFRβ as a non-invasive biomarker for prediction of liver fibrosis in diabetic MAFLD patients. Most MAFLD patients in our study were females, and MAFLD subjects with T2DM were older than the nondiabetics as reported previously [31–33]. The waist circumference, waist/hip circumference ratio, and BMI were significantly higher in MAFLD subgroups than controls, as previously confirmed [34–39]. Similarly, visceral fat, fat percentage, and fat mass values were significantly higher in MAFLD subgroups compared to controls, in line with several previous studies [40–44]. Also, visceral fat as well as metabolic syndrome features were significantly higher in diabetic compared to nondiabetic MAFLD patients, in agreement with previous reports [45, 46].

PDGFRβ in our study was significantly higher in both MAFLD subgroups compared to controls. It was reported that PDGFRβ expression is extremely decreased in the healthy liver but markedly increased during liver injury [47]. We found significantly higher AST and ALT levels in MAFLD subgroups than controls, as previously reported [48, 49]. Serum albumin was significantly lower in diabetic MAFLD patients than controls, which can be due to the effect of T2DM on the kidney [50].

MAFLD subgroups had significantly higher total cholesterol, LDL, and triglycerides than controls. Also, lower HDL was observed in MAFLD subgroups compared to controls that reached statistical significance when comparing diabetic MAFLD and control groups. This pattern of dyslipidemia has been closely linked to MAFLD [51]. Moreover, diabetic MAFLD patients had significantly higher total cholesterol and LDL than the nondiabetic MAFLD group. Similar results were detected by previous studies [32, 52].

Liver steatosis assessed by MRI in our study was significantly higher in diabetic than non-diabetic MAFLD patients. A previous study also showed that hepatic steatosis was significantly higher in diabetic patients [53]. Diabetes is the most important risk factor for the development and progression of hepatic steatosis as well as liver fibrosis. Liver-related outcomes in patients with MAFLD are influenced by the advanced stage of fibrosis and not steatosis [54]. We assessed non-invasive scores of liver fibrosis in diabetic patients with MAFLD. AST/ALT ratio, APRI, King’s score, MAFLD fibrosis score, FIB-4, hepamet score, and PDGFRβ + FIB-4 were significantly higher in diabtetic MAFLD compared to non-diabetic patients. **Drolz et al.** concluded that non-invasive scoring systems are useful predictors of liver fibrosis in MAFLD subjects [55]. However, **Bertot et al.** found that non-invasive scoring systems are less accurate at predicting fibrosis progression in diabetic MAFLD patients [32]. Kings score is not yet validated in MAFLD subjects. It was initially evaluated for diagnosis of cirrhosis in patients with hepatitis C infection [25].

We also measured liver stiffness in MAFLD patients using MRE. Liver stiffness was significantly higher in diabetic than nondiabetic MAFLD patients. We found a significantly higher percentage of patients having significant liver fibrosis (≥ F2) in the diabetic MAFLD group compared to the nondiabetic group (40% vs. 17.5%). **Park et al.** found that the risk of significant fibrosis was higher in the diabetic MAFLD cohort than in the non-glucose-intolerant group [56].

Liver stiffness by MRE in our study correlated positively with APRI, King’s score, NAFLD fibrosis score, FIB-4, and Hepamet fibrosis score in total MAFLD patients. **Fallatah et al.** reported a significant positive association between liver stiffness measurements by fibroscan and AST/ALT ratio, APRI, and FIB-4 indices [57]. We also found a positive correlation between LSM and FIB-4 score in both diabetic and nondiabetic MAFLD patients, while APRI was positively correlated with LSM in nondiabetic MAFLD only. Similar results were reported by **Shaji et al.** [58]. **Saran et al.** found that the APRI score doesn’t correlate with imaging evidence of fibrosis in diabetic MAFLD subjects [59].

In our study, the sensitivity, specificity, PPV, and NPV of the FIB-4 score to predict significant liver fibrosis (≥ F2) in diabetic MAFLD patients were 80%, 73.33%, 66.7%, and 84.6%, respectively, at a cutoff level > 1.17 (AUC = 0.719, CI = 0.568–0.870, *P* = 0.009). In nondiabetics, they were 71.43%, 75.76%, 38.5%, and 92.6%, respectively, at a cutoff level > 0.96. Using previously validated cutoff levels of FIB-4 (1.45 and 3.25), used to predict advanced liver fibrosis (< F3) in viral hepatitis, in MAFLD patients to predict significant liver fibrosis (*≥* F2) showed unsatisfactory performance. A meta-analysis of 36 studies revealed that the diagnostic accuracy of FIB-4 was relatively high in fibrosis stages ≥ F3 [60]. The most extensively validated non-invasive scores for determining a patient’s risk of advanced fibrosis in MAFLD are the FIB-4 score and the NAFLD fibrosis score. These tests are considered clinically valuable tools. Nevertheless, in at least 30% of the cases, these scores are suggestive of “indeterminate” ranges [61]. They have lower specificity in elderly patients, and new cutoff points have been suggested for those who are 65 years of age or older [62]. Moreover, these tests were not designed as screening tools but rather as part of a cohort study where severe fibrosis was more common. As a result, more specialized diagnostic tests must be performed in succession. Additionally, there might be variations in diagnostic performance between cohorts with and without diabetes, as well as possible racial disparities that could affect test accuracy [63].

We found that the performance of PDGFRβ as a non-invasive marker to predict significant liver fibrosis in diabetic MAFLD was superior to the FIB-4 score. The same finding was detected in a previous study by **Lambrecht et al.** that assessed serum PDGFRβ’s usefulness in predicting significant liver fibrosis across different etiologies (viral, alcoholic, and non-alcoholic liver disease in T2DM). They found that the predictive function of serum PDGFRβ is independent of disease etiology. Their study included 67 European MAFLD patients who were all diabetics [17]. Our study confirmed the diagnostic accuracy of serum PDGFRβ in Egyptian MAFLD patients in both diabetics and nondiabetics. A division of MAFLD subjects based on T2DM identified a strong discriminative ability of PDGFRβ for significant liver fibrosis. The performance of PDGFRβ was the best in diabetic MAFLD patients (AUC 0.907), with sensitivity, specificity, PPV, and NPV of 85%, 93.33%, 89.5%, and 90.3%, respectively, at a cutoff > 2.54, while its AUC in the **Lambrecht et al.** study was 0.6406. This may be due to differences in demographic criteria, number of studied populations, and liver stiffness assessment by acoustic radiation force impulse elastography [17].

PDGFRβ sensitivity, specificity, PPV, and NPV in non-diabetic MAFLD subjects were 85.71%, 51.52%, 27.3%, and 94.4%, respectively, at a cutoff value > 1.59, while the values were 57.14%, 87.88%, 50%, and 90.6% using the same cutoff (> 2.54) used in the diabetic MAFLD group. The sensitivity, specificity, PPV, and NPV of PDGFRβ + FIB-4 in diabetic MAFLD were all 100%, indicating that adding PDGFRβ to the FIB-4 score enhanced its ability to identify severe liver fibrosis in these subjects. Factors predicting significant liver fibrosis in the diabetic MAFLD group were PDGFRβ, smoking, visceral fat, and FIB-4 score, while PDGFRβ was the only independent predictor. Therefore, in diabetic patients with MAFLD, particularly individuals with FIB-4 intermediate ranges or elderly patients, the use of PDGFRβ or its incorporation with the FIB-4 score can improve significant fibrosis detection and prevent the need for an invasive liver biopsy or more costly imaging techniques. Similarly, serum PDGFRβ could complement or improve the performance of other non-invasive scores as well as non-invasive imaging modalities. PDGFRβ can also be useful in treatment monitoring of MAFLD patients since it is overexpressed during liver injury and its circulating levels can reveal information about the degree of liver fibrosis. Treatment options for the management of individuals with MAFLD can lead to resolution of hepatic fibrosis, during which hepatic stellate cells undergo apoptosis, become senescent, or revert to an inactive state with diminished PDGFRβ production [64].

Egypt has one of the highest prevalences of MAFLD at approximately 47.5%, with 56.7% having fibrosis. Given that the severity of fibrosis is the major determinant of both hepatic-related outcomes and mortality, patients with significant fibrosis need the closest monitoring. Identification of patients at risk is necessary for treatment decisions. The Egyptian guidelines for the diagnosis and management of MAFLD recommend using simple noninvasive biomarkers and scores of fibrosis for assessing disease severity and monitoring disease progression and treatment response. However, their cut-offs need to be further validated in Egyptian cohorts, and liver biopsy is still required in some cases, particularly in patients with indeterminant (gray) range scores [65]. Using PDGFRβ as a biomarker could improve non-invasive assessment of liver fibrosis in this patient population by increasing the accuracy of prediction and minimizing the gray zone.

The strengths of our study included that a strong case-control design was used with diabetic MAFLD patients, non-diabetic MAFLD patients, and healthy controls, enabling accurate comparison and validation of PDGFRβ as a biomarker. Also, the findings were validated using a variety of statistical methods, including multivariate logistic regression, increasing the dependability of the findings. Furthermore, this study distinguishes itself from previous studies on noninvasive markers for liver fibrosis by focusing solely on diabetic MAFLD patients. Previous studies have shown that noninvasive diagnostics intended for non-diabetic populations often work poorly in diabetic individuals. This work fills this gap by demonstrating that PDGFRβ more accurately predicts severe liver fibrosis in diabetic MAFLD patients, especially when combined with the FIB-4 score. Our findings have important clinical implications since they provide a more reliable non-invasive diagnostic tool for liver fibrosis in diabetic MAFLD patients, overcoming the limits of current biomarkers in these patients and reducing the need for invasive liver biopsies.

Nevertheless, our study has several limitations. A relatively small number of the studied subjects may impact the statistical power, and they were recruited from a single center and hence might not be truly representative of the broader population. Also, the study focused on PDGFRβ in comparison to only the FIB-4 score. A comparative analysis is still needed with other established biomarkers of liver fibrosis, such as alpha-smooth muscle actin (α-SMA), collagen, and integrins. Besides, we measured serum PDGFRβ at one timepoint, which may not accurately reflect the dynamic nature of fibrosis and its response to treatment. Additional patient monitoring is still needed to detect PDGFRβ correlation with long-term clinical outcomes. Conducting long-term, multi-center studies on a large population, including diabetic and nondiabetic subjects in a variety of liver fibrosis etiologies, along with cost-effectiveness analysis should be taken into consideration for subsequent research. The lack of a liver biopsy, which is regarded as the gold standard for determining the degree of disease activity and liver fibrosis, was another drawback of the current study. We used MRI and MRE to evaluate the liver steatosis degree and the liver stiffness, respectively, being more accurate to overcome the limitations of other non-invasive methods. A more conclusive evaluation of liver fibrosis would also be possible with the inclusion of a liver biopsy in future research.

## Conclusions

PDGFRβ levels were significantly higher in both diabetic and non-diabetic MAFLD patients compared to controls. This suggests that PDGFRβ is a reliable indicator of liver fibrosis severity. The sensitivity, specificity, PPV, and NPV of PDGFRβ to predict significant liver fibrosis in diabetic MAFLD patients were 85%, 93.33%, 89.5%, and 90.3%, respectively, at a cutoff > 2.54. These values indicate a high diagnostic accuracy. The combination of PDGFRβ at a cutoff > 2.54 and FIB-4 at a cutoff > 1.17 resulted in 100% sensitivity, specificity, PPV, and NPV for predicting significant liver fibrosis in diabetic MAFLD patients. Such findings may indicate the reduced need for invasive liver biopsies. Additionally, PDGFRβ could complement other non-invasive scores and imaging modalities, providing a more comprehensive assessment of liver fibrosis.

## Data Availability

All data generated or analyzed during this study are included in this published article.

## Abbreviations

AST/ALT: Aspartate transaminase/Alanine transaminase
AUC: Area under the curve
BIA: Bioelectrical impedance analysis
BMI: Body mass index
CI: Confidence interval
ELISA: Enzyme-linked immunosorbent assay
FI: fat indices
FIB-4: Fibrosis-4
HbsAg: Hepatitis B surface antigen
HCV: Hepatitis C virus
HDL-C: High-density lipoprotein cholesterol
HSCs: Hepatic stellate cells
kPa: Kilopascal
LDL: Low-density lipoprotein
LSM: Liver stiffness measurement
MRE: Magnetic resonance elastography
MRI: Magnetic resonance imaging
MAFLD: Metabolic-associated fatty liver disease
MASH: Metabolic-associated steatohepatitis
NFS: NAFLD fibrosis score
NPV: Negative predictive value
PDGFRβ: Platelet-derived growth factor receptor-β
PPV: Positive predictive value
ROIs: Regions of interest
SI: Signal intensity
T2DM: Type 2 diabetes mellitus
